# Chemical Modification of Softwood Kraft Lignin with Succinic Acid

**DOI:** 10.1021/acsomega.4c03127

**Published:** 2024-12-19

**Authors:** Gabriel Resende, Gustavo D. Azevedo, Felipe Souto, Veronica Calado

**Affiliations:** Programa de Engenharia de Processos Químicos e Bioquímicos, Escola de Química, Centro de Tecnologia, Universidade Federal Do Rio de Janeiro, Av. Athos da Silveira Ramos, 149, Bloco E, Ilha do Fundão, Rio de Janeiro 21941-909, Brasil

## Abstract

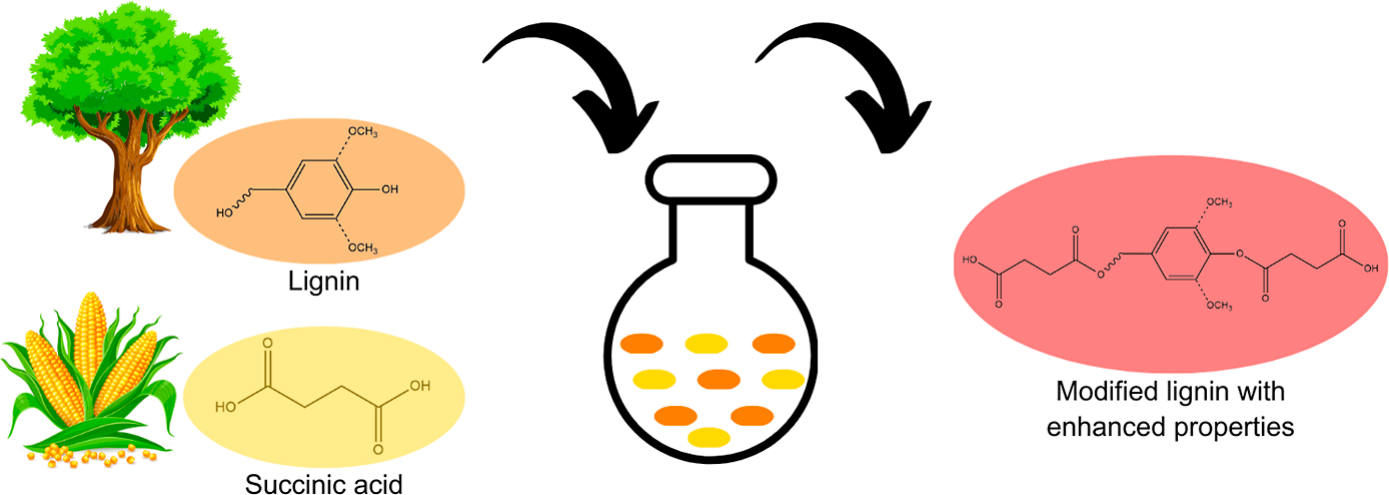

This work explored the chemical modification of lignin with succinic acid for the first time. Temperature is crucial for the process, reducing reaction time and increasing conversion. In particular, at 160 °C for five h with 0.13 mol of imidazole and 0.35 mol of succinic acid, the reaction showed the most significant reduction in glass transition temperature (from 183.3 to 118.5 °C) and a decrease in the polydispersity index (from 11.31 to 8.96) and yielded 0.73 mmol g^–1^ of new succinic acid groups. These results underscore the critical role of reaction conditions in modifying lignin properties, potentially expanding its application range and enhancing its value.

## 1 Introduction

Woody and nonwoody lignin molecules primarily comprise three canonical radicals: *p*-hydroxyphenyl, guaiacyl, and syringyl radicals, which are derived from monolignols.^1^ These monolignols are randomly linked, and the proportion of these units in the chemical structure of lignin, along with the extraction process, influences its properties.^2^ For instance, softwood kraft lignin exhibits greater reactivity because of a higher abundance of guaiacyl radicals, which provides less steric hindrance. This characteristic makes this specific type of lignin particularly advantageous for developing large-scale kraft lignin-based products.^3^

Lignin is undoubtedly one of the most underutilized products generated during biomass processing, primarily serving as an energy source for pulp mills.^4^ The limitations in its applications arise from variations in its chemical structure, which can differ depending on the type of plant and extraction method. These variations significantly influence lignin’s mechanical and thermal properties.

As biorefineries develop worldwide, numerous strategies have been proposed to utilize lignin as a substitute for petroleum derivatives, as a source of chemicals, and as an additive in advanced materials.^4−11^ For example, lignin is a viable source for extracting vanillin, a pivotal chemical compound extensively employed in the food industry, boasting an annual production rate of 20,000 tons, with 15% derived directly from lignin.^6^ Silva et al. (2009) and Rodrigues Pinto et al. (2012) have elucidated an integrated process for synthesizing vanillin alongside lignin-based polyurethanes, leveraging kraft lignin, thereby broadening the scope of applications for this versatile molecule.^12,13^

Nevertheless, certain modifications are sometimes required to overcome the limitations of lignin and to enhance its properties for specific applications. The literature reports various applications of modified kraft lignin, including dispersants, adhesives, and other uses.^14^ Utilizing lignin as a biobased material for advanced and sustainable manufacturing is an important goal, particularly in enhancing the correlations among structure, properties, and performance. Consequently, numerous studies have investigated modifications to lignin that may yield significant advancements in industrial applications.

A notable example is the development of lignin-based resins. Subbotina et al. (2024) maleated lignin with maleic anhydride in 4-dimethylaminopyridine, obtaining a degradable thermoset. This reaction introduced new functionalities to the lignin, potentially providing suitable thermosets with tetra-functional thiols.^15^ Scarica et al. (2018) functionalized lignin with succinic anhydride to create thermosetting polyester coatings. This succinylation process improved cross-linking in polyester-coating applications. Before the derivatization, kraft softwood lignin was fractionated using tetrahydrofuran (THF), where the soluble fractions underwent esterification in the presence of 1-methylimidazole and succinic anhydride.^16^ Similarly, Bellinetto et al. (2024) capitalized on the advantages of esterified lignin as an accelerator for cross-linking curing reactions, employing a succinylation reaction in the formulation of phenol-formaldehyde resins.^17^ The authors achieved a high-performance phenolic resin by adhering to methodological principles similar to those of Scarica et al. (2018).

Regardless of significant advancements in the use of lignin for this purpose, the reactions mentioned above involve toxic and hazardous chemicals, compromising the sustainability of the process. Furthermore, many researchers promote lignin derivatives as examples of green procedures, overlooking that the reagents used to produce these derivatives can negatively impact the environment and human health. Therefore, there is a need for reactions that can achieve the desired modifications with low toxicity and minimal environmental impact. Given these challenges, alternative reagents, such as succinic acid, emerge as promising options.

Succinic acid (SA) is a dicarboxylic acid with significant potential as a future chemical platform derived from renewable sources. This compound can be produced by sugar fermentation and serves as a raw material for synthesizing various molecules, including polyesters, polyamides, pyrrolidones, and lactones. Unlike anhydride succinic, succinic acid is nontoxic and recognized as a safe food additive in the Generally Recognized As Safe database maintained by the Food and Drug Administration. It is also already available as a byproduct in the adipic-acid-manufacturing industry.^18^ These advantages position SA as a preferred reagent for lignin modification over traditional anhydride.

Anhydrides and acid chlorides are commonly used for esterification reactions. While anhydrides provide a similar chemical environment to acyl chlorides; acid chlorides are moisture sensitive, commercially less available, and can produce unpredictable byproducts.^19^ Although these reagents are preferred in many cases, they may not always be ideal, especially when they are scaled up for commercial applications. In such instances, the condensation reaction between a carboxylic acid and an alcohol is often favored. To the best of the authors’ knowledge, while SA has been evaluated as an additive and plasticizer for lignin applications, it has not been studied as a derivatizing or esterifying reagent. That strengthens the objective of investigating this pathway once lignin research into value-added products is exponentially advancing, and political initiatives drive sustainable developments.

Based on the previous discussion, this study aims to modify lignin with SA chemically, leveraging its structure and potential availability from renewable sources. The goal is to evaluate the succinate reaction on lignin using an eco-friendly approach, with SA as the modifier and dimethyl sulfoxide (DMSO) as a greener solvent. This approach is novel and has not been previously reported, as far as these authors know. A factorial experimental design was employed to evaluate the statistical influence of critical factors, including temperature, reaction time, amount of catalyst, and SA quantity, within defined ranges. Moreover, the study aligns with sustainability principles by utilizing sustainable raw materials (lignin and succinic acid) and generating water as the sole byproduct, which can be repurposed in the separation and purification processes of the reaction.

## 2 Materials and Methods

### 2.1 Materials

Pine kraft lignin (*M*_w_ = 6647 g·mol^–1^) was provided by Klabin (Paraná, Brazil). Imidazole (99.0 wt %), succinic acid (SA, 99.0 wt %), dimethyl sulfoxide (DMSO, 99.9 wt %), 2-chloro-4,4,5,5-tetramethyl-1,3,2-dioxaphospholane (99.9 wt %), chromium(III) acetylacetonate (99.99 wt %), *endo*-*N*-hydroxy-5-norbornene-2,3-dicarboximide (99 wt %), pentafluorobenzaldehyde (98 wt %), deuterated dimethyl sulfoxide (99.9 wt %), deuterated pyridine (99.9 wt %), and deuterated chloroform (99.9 wt %) were provided by Merck (Darmstadt, Germany). All chemicals were used as received without prior purification steps.

### 2.2 Methods

#### 2.2.1 Lignin–Succinic Acid Derivatization

Lignin–succinic acid (L–SA) was obtained by dissolving lignin in DMSO at a concentration of 10% (w/v). The reaction mixture was prepared by dissolving the experimental amount of lignin and succinic acid (as detailed in Table 1) in DMSO under vigorous stirring. The mixture was poured into a 250 mL three-necked rounded flask equipped with a thermocouple. The flask was settled on a mineral oil heating bath, and the heating was provided using a hot plate with magnetic stirring and temperature control.

**Table 1:** Full Factorial Experimental Design for the Synthesis of L–SA

					coded levels			
experiment	temperature (°C)	imidazole (mol)	time (h)	SA (mol)	X_1_	X_2_	X_3_	X_4_
R1	160	0.13	15.0	0.69	+1	+1	+1	+1
R2	160	0.13	15.0	0.35	+1	+1	+1	–1
R3	160	0.04	5.0	0.35	+1	–1	–1	–1
R4	160	0.13	5.0	0.35	+1	+1	–1	–1
R5	120	0.04	5.0	0.35	–1	–1	–1	–1
R6	120	0.13	15.0	0.35	–1	+1	+1	–1
R7	120	0.13	5.0	0.35	–1	+1	–1	–1
R8	120	0.04	15.0	0.35	–1	–1	+1	–1
R9	160	0.13	5.0	0.69	+1	+1	–1	+1
R10	120	0.13	15.0	0.69	–1	+1	+1	+1
R11	120	0.04	15.0	0.69	–1	–1	+1	+1
R12	120	0.04	5.0	0.69	–1	–1	–1	+1
R13	120	0.13	5.0	0.69	–1	+1	–1	+1
R14	160	0.04	15.0	0.69	+1	–1	+1	+1
R15	160	0.04	5.0	0.69	+1	–1	–1	+1
R16	160	0.04	15.0	0.35	+1	–1	+1	–1
R17	140	0.09	10.0	0.52	0	0	0	0
R18	140	0.09	10.0	0.52	0	0	0	0
R19	140	0.09	10.0	0.52	0	0	0	0

Once the system reached the experimental temperature (as detailed in Table 1), imidazole was added to the flask, initiating the reaction. At the end of the reaction, the product was precipitated using deionized water, and L–SA was filtrated under vacuum until a neutral pH of the filtrate was achieved. Finally, the modified lignin was dried in a vacuum oven at 60 °C for 24 h and stored in a Schott flask.

**Figure 1:**
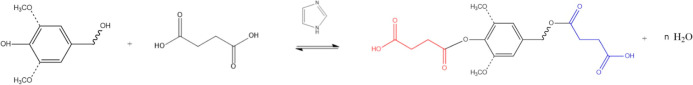
Reaction scheme for the modification of lignin with succinic acid. The syringyl radical was represented generically. Additionally, modification can occur at both phenolic and aliphatic hydroxyl groups along the propane chain of the molecule.

The reaction scheme is shown in Figure 1.

#### 2.2.2 Experimental Design

The influence of temperature (X_1_), catalyst concentration (X_2_), reaction time (X_3_), and SA concentration (X_4_) on SA content on L–SA was evaluated by a 2^4^ full factorial experimental design, leading to 16 experiments (R1–R16) and three replicates at the central point (R17–R19), as shown in Table 1.

#### 2.2.3 L–SA Characterizations

All lignin samples were characterized by using dry aliquots from each sample. The drying was performed in an oven at 60 °C for 24 h and stored in a desiccator until analysis.

Fourier transform infrared spectroscopy (FTIR) analysis was performed to identify groups from SA present in the L–SA. The analyses were conducted in a Frontier spectrophotometer (PerkinElmer, Waltham, Massachusetts, EUA) equipped with a germanium crystal universal ATR accessory. Thirty-two scans per sample were performed, with a spectral resolution of 4 cm^–1^. All data spectra were processed and adjusted using PerkinElmer Spectrum IR software, version 10.6.2. Spectra were normalized by dividing the intensity values of each data point by the highest intensity peak.

Nuclear magnetic resonance spectroscopy (NMR) was performed on samples prepared as previously described in the literature.^20,21^ For quantitative ^1^H NMR, approximately 20 mg of lignin was dissolved in 600 μL of DMSO-*d*_6_, along with 22 μL of the internal standard (IS) (2,3,4,5,6-pentafluorobenzaldehyde). A total of 128 scans were recorded with a relaxation delay of 10 s. For ^31^P NMR spectroscopy, approximately 50 mg of lignin was dissolved with the relaxation agent (chromium(III) acetylacetonate, 2.5 mmol L^–1^), the IS (*endo*-*N*-hydroxy-5-norbornene-2,3-dicarboximide, 0.015 mmol), and the phosphorylating agent (2-chloro-4,4,5,5-tetramethyl-1,3,2-dioxaphospholane, 0.78 mmol) in a mixture of pyridine-d_5_ and chloroform (1.6:1, v/v). For this analysis, 512 scans were collected with an acquisition time of 1.0 s and a relaxation delay of 5.0 s. All analyses were conducted in a Bruker AV-400 NMR spectrometer (Massachusetts), operating at 400 MHz and at a temperature of 25 °C. Both ^1^H and ^31^P NMR data were processed using MestReNova software, version 12.0. The ^1^H NMR spectra were Fourier transformed, phased, and calibrated using a 2,3,4,5,6-pentafluorobenzaldehyde signal as a reference (10.28 ppm), and the baseline was corrected using a polynomial fit (polynomial order 1). The ^31^P NMR spectra were Fourier transformed, phased, and calibrated using a water-derivatized product (132.2 ppm). The baseline was corrected using a polynomial fit (polynomial order 1).

Size exclusion chromatography (SEC) was based in Sulaeva et al. (2017) methodology and was used to estimate the number-average molar weight (*M*_n_), weight-average molar weight (*M*_w_), and polydispersity index (PDI) of lignin and L–SA.^22^ The analyses were carried out in an Acquity UPLC H-Class (Waters Corporation, Milford, Massachusetts, USA) equipped with Acquity APC XT 450, 200, 125, and 45 Å (2.5 μm particle size), a precolumn, and an RI detector. The temperatures of the column and detector were 90 and 40 °C, respectively. Calibration was performed using polystyrene sodium sulfonate standards with molecular weights ranging from 246 to 258,000 Da and PDI ≤ 1.20. Samples were prepared with a concentration of 3.0 mg mL^–1^, dissolving in a 0.5% DMSO/LiBr solution and stirring in an orbital shaker agitator at 150 rpm for 90 min. Then, the samples were filtrated using a Teflon filter with a pore size of 0.22 μm. The injection volume was 3.0 μL with an operating flow rate of 0.5 mL min^–1^.

Dynamic mechanical analysis was performed to determine the glass transition temperatures (*T*_g_) of both kraft lignin and L–SA. *T*_g_ is an important parameter to measure the segmental mobility of polymer chains and, therefore, the processability of polymeric materials.^23^ This property is obtained from the peak of the tan δ thermogram (the ratio between the loss modulus and storage modulus) in a dynamic mechanical analysis. The analyses were carried out in a DMA Q800 (TA Instruments, USA) in a frequency of 1 Hz in stretch and 1% strain, from room temperature to 220 °C at 3 °C min^–1^. The samples were placed in a rectangular powder sample holder of dimensions of 35.0 mm length × 12.8 mm width × 3.2 mm thickness. The sample holder was mounted in the instrument in a 3-point bend clamp of dimensions 15 mm length × 15 mm width × 7 mm thickness.

The thermal stability of modified lignin was determined by a thermal gravimetric analysis. Derivative thermogravimetry (DTG) was carried out on a Shimadzu TGA-50 instrument (Shimadzu, Kyoto, Japan) under a nitrogen atmosphere at a heating rate of 10 °C min^–1^. Temperature scan was evaluated from ambient temperature to 600 °C.

## 3 Results and Discussion

**Figure 2:**
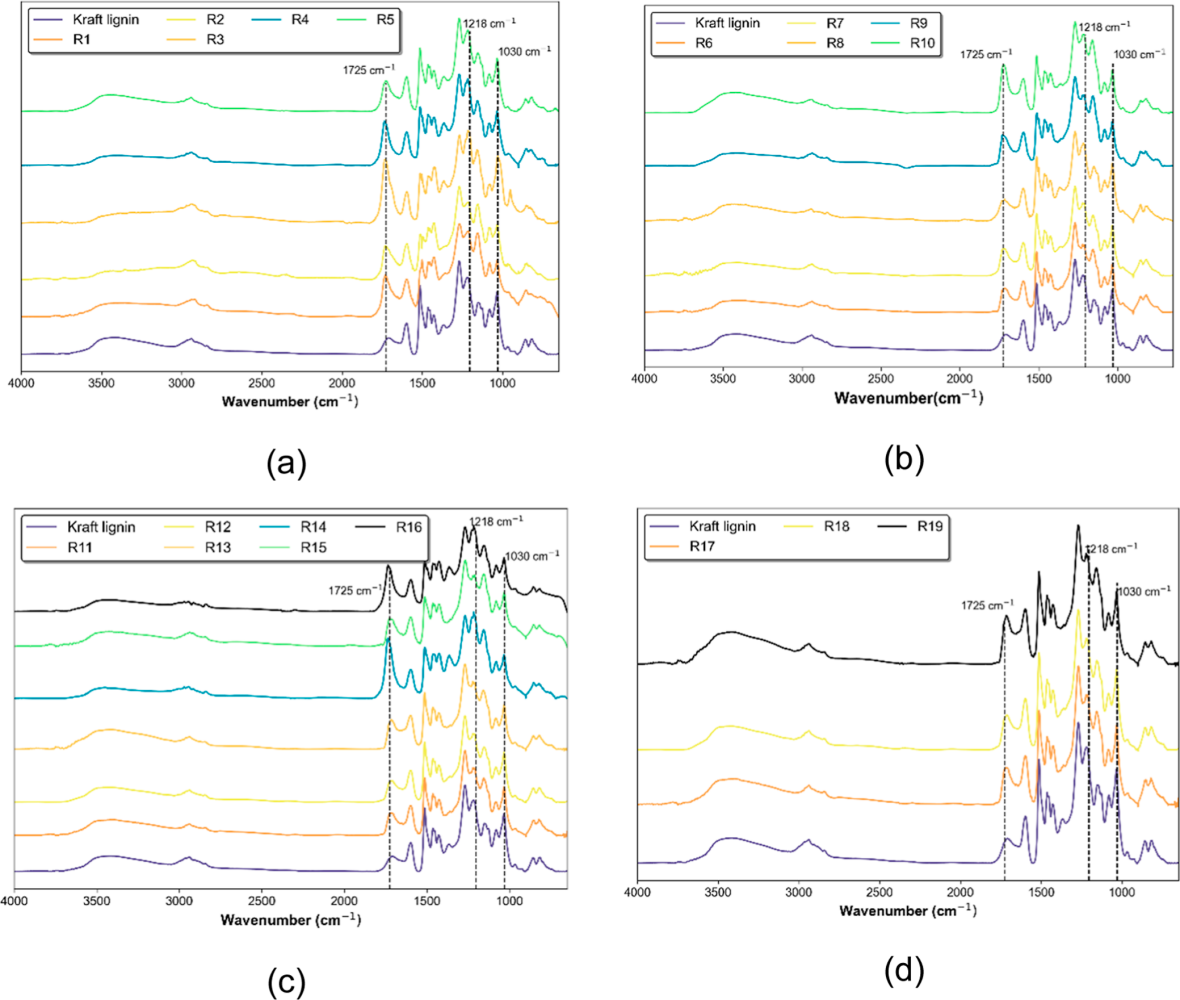
FTIR spectra of kraft lignin and L–SA. (a) R1–R5; (b) R6–R10; (c) R11–R16; (d) R17–R19.

FTIR spectra of kraft lignin and all L–SA samples listed in Table 1 are presented in Figure 2. First, it is important to identify and describe the bands observed in the kraft lignin spectra. These bands are detailed in Table 2.^24−28^

**Table 2:** Description of the Bands Assigned to Kraft Lignin in FTIR Spectra

assigned FTIR band	wavenumber (cm^–1^)
hydroxyl (OH) stretch	3494
C–H stretch from methyl and methylene groups	2947
C=O stretch from ester	1725
aromatic ring vibration associated with C=O	1598
aromatic ring vibration	1514
asymmetric C–H deformation in methyl and methylene groups	1465
aromatic ring vibration associated with C–H deformation in the plane	1427
guaiacyl units vibration with C=O	1273
C–C, C–O, and C=O stretch mainly from condensed guaiacyl units	1218
aromatic deformation in the plane, primary alcohol C–H deformation, and unconjugated C=O stretch	1156
C–O deformation mainly in primary alcohol, aliphatic ethers, and C–H in aromatic ring	1030
C–H deformation out of the plane of the hydrogens from *p*-hydroxyphenyl units	824

The spectrograms qualitatively indicate that the reaction between lignin and succinic acid largely preserves the lignin structure under all of the tested conditions. However, an increase in intensity and a change in the profile of specific spectral regions, namely, at 1725, 1218, and 1030 cm^–1^, were observed. These bands correspond primarily to C=O stretching, C–O stretching, and C–O deformation, respectively.^28^ Such changes can be attributed to the esterification of lignin with succinic acid but may also indicate the presence of residual succinic acid, which would overlap with similar stretching and deformation bands.

The spectrum of sample R3 is particularly noteworthy as it presents the most prominent peak at 1725 cm^–1^, corresponding to the carbonyl (C=O). Because of uncertainties regarding the extent of the reaction, ^1^H and ^31^P NMR spectroscopy were employed to provide quantitative results and confirm the reaction.

**Figure 3:**
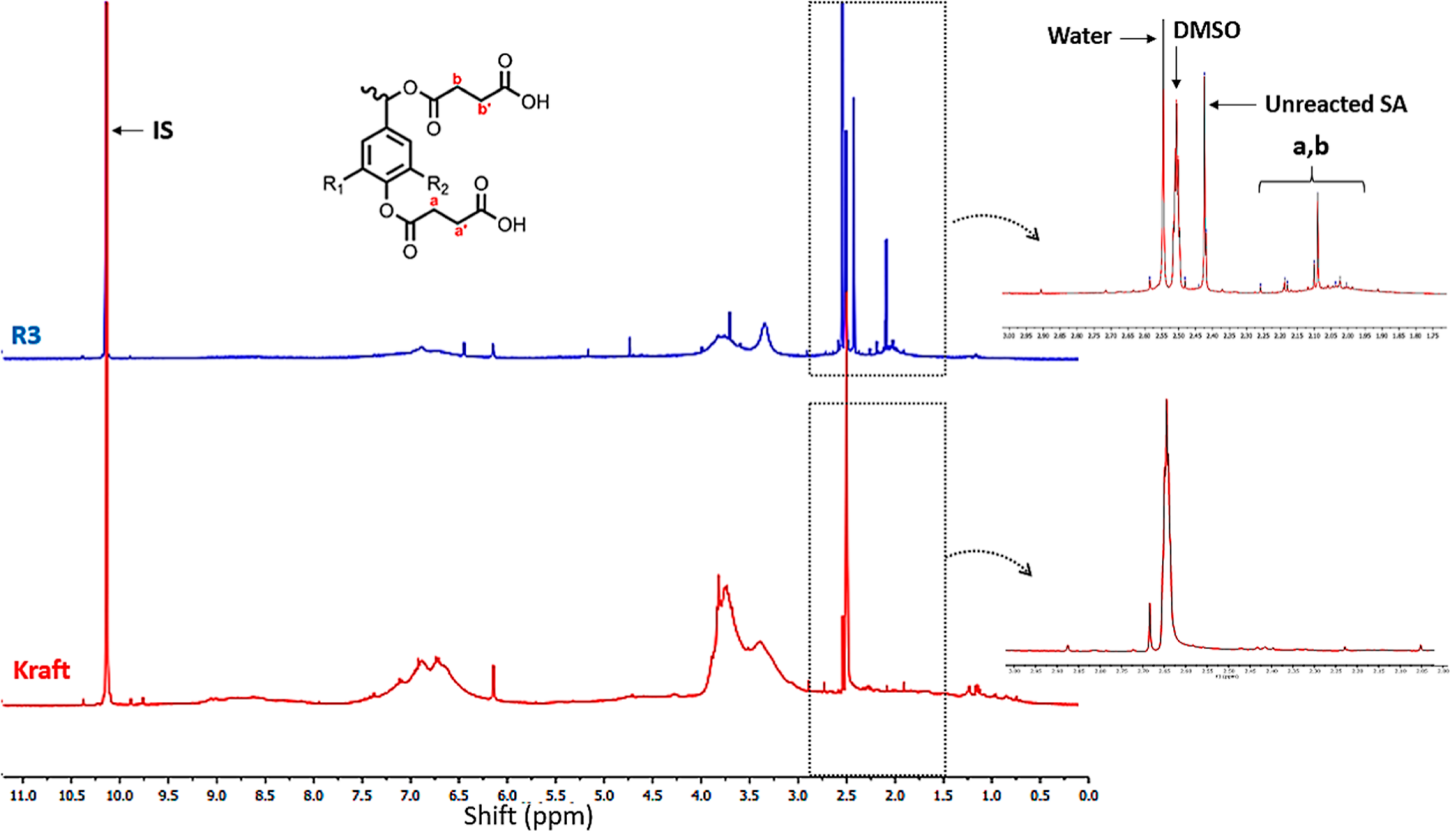
^1^H NMR spectra of kraft lignin and R3. IS corresponds to the internal standard.

^1^H NMR analyses were performed on all samples, with the spectra for kraft and modified lignin (R3) shown in Figure 3. Additional spectra can be found in the Supporting Information. The chemical modification of lignin with succinic acid is evidenced by the appearance of new signals in the 1.7 ppm to 2.2 ppm region, which are attributed to aliphatic and phenolic acetyl groups. The newly introduced succinic acid groups (N_SA_) quantified in the lignin structure were based on this region and an IS.^21^

Despite these new signals, a sharp peak at 2.42 ppm was observed in all modified samples, which is associated with free succinic acid and confirmed by MestReNova predictions.^29^ This peak can be used to quantify the impurity of the obtained samples and correlate with the qualitative FTIR spectra (free SA content values are provided in the Supporting Information). That is crucial for interpreting the qualitative FTIR results accurately. As mentioned earlier, the R3 sample exhibited the highest C=O intensity among all of the observed samples, which could suggest a higher incorporation of succinic acid in the samples.

**Figure 4:**
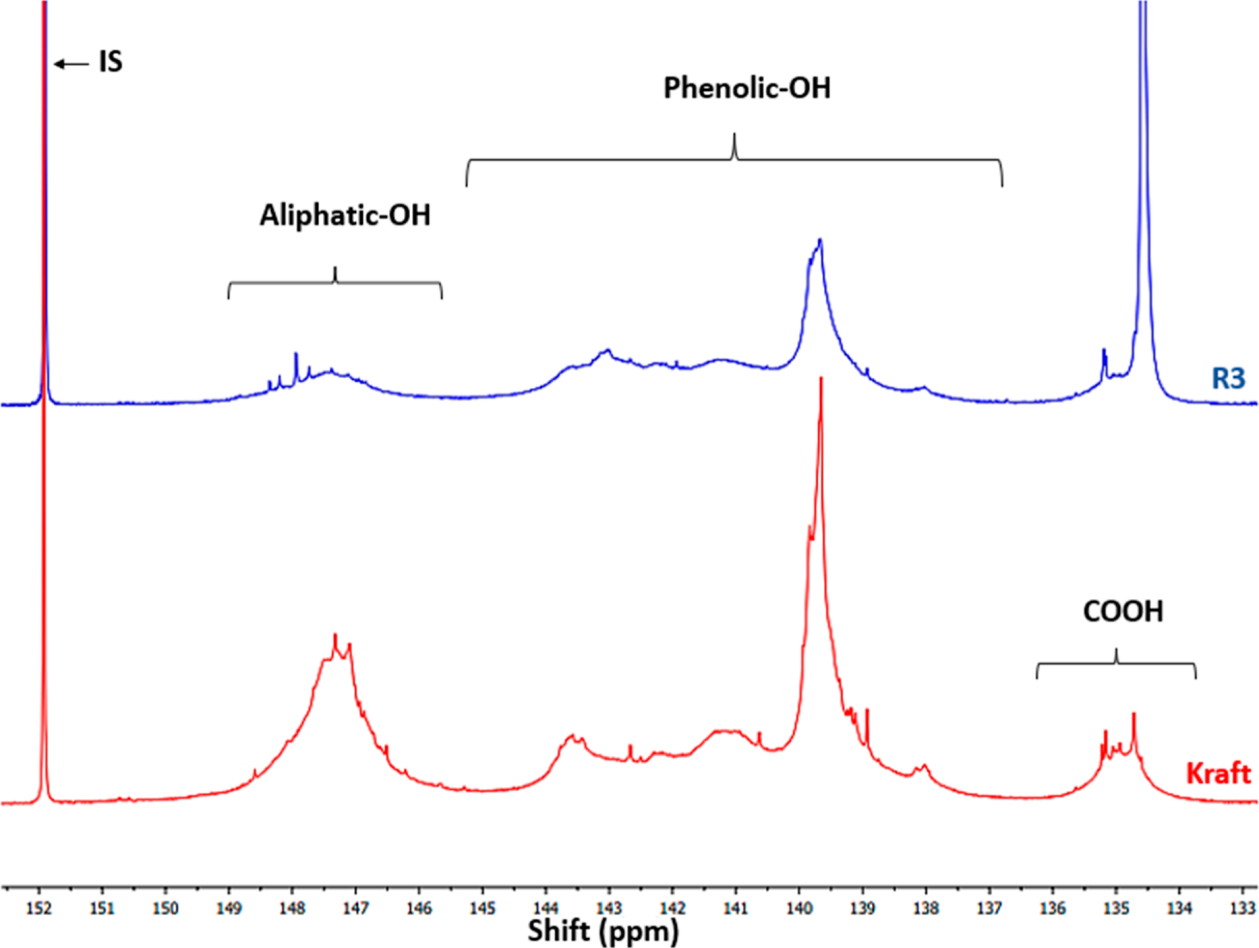
^31^P NMR spectra of kraft lignin and R3.

For the ^31^P NMR analyses, R3 was selected arbitrarily to confirm the chemical modification of lignin with succinic acid (Figure 1) and attempted to provide extra information about the mechanism involved. The spectra of kraft lignin and modified lignin (R3) are shown in Figure 4. The regions corresponding to the aliphatic, phenolic, and carboxyl hydroxyl groups (Ali–OH, Ph–OH, and COOH, respectively), as well as the IS, were integrated as described in the literature.^20,30^ Additionally, the integrated areas and concentrations of the mentioned groups are provided in Table 3.

**Table 3:** Hydroxyl Content of Kraft Lignin and R3

sample	[OH] (mmol/g lignin)				conversion (%)		
	Ali–OH	Ph–OH	COOH	total^a^	Ali–OH	Ph–OH	overall^a^
kraft lignin	2.84	5.62	0.73	8.46			
R3	0.61	2.57	1.38	3.19	78.2%	54.2%	62.2%

a Notes: Total refers to Ali–OH and Ph–OH’s sum of [OH] in mmol/g lignin. Overall refers to the percentage conversion of the [OH] content based on the kraft lignin.

The neat lignin spectrum obtained presented a typical profile characteristic of softwood lignin, as expected. The esterification of both aliphatic and phenolic hydroxyls in lignin resulted in an apparent decrease in their content, accompanied by an increase in the carboxyl content (Figure 4 and Table 3). That can be explained by the fact that succinic acid is a dicarboxylic acid, with one carboxyl group reacting with the lignin hydroxyl, while the other may remain unreacted. However, this hypothesis can lead to the erroneous assumption that the decrease in esterified phenol or alcohol in the lignin corresponds directly to increased carboxyl (COOH) content. Three important considerations must be taken into account: (1) both carboxyl groups of succinic acid may react in different ways, such as through cross-linking with other lignin hydroxyl groups or undergoing further reactions; (2) the OH content of R3 is measured in mmol per gram of derivatized lignin, where the mass of neat lignin increases because of the addition of the mass of newly introduced groups.^21^ The change in sample mass leads to an underestimation of hydroxyl content when calculated by ^31^P NMR, resulting in an overestimation of the conversion of OH content. (3) The COOH amount contains derivatized groups and free SA trapped/associated with the samples.

Despite the overestimation of conversion based on mass differences, this does not change the fact that the aliphatic hydroxyls reacted more than the phenolic ones. That is reasonable as Fisher esterification involves the reaction of alcohols with carboxylic acids. Phenolic hydroxyls are more acidic and may react only because of the presence of a basic catalyst applied to the reaction medium. These findings are consistent with those obtained by Liu et al. (2019) but contrast with the results of Buono et al. (2016).^21,31^ The former propionated kraft lignin through Fischer esterification, reacting lignin with propionic acid, which is more likely to resemble the present investigation. The latter esterified lignin with anhydride in a highly alkaline medium using both solvent (pyridine) and catalyst as base reactants, thus favoring the deprotonation of phenols into phenolate nucleophiles.

Table 4 summarizes results obtained for inserted succinic acid (N_SA_), glass transition temperature (*T*_g_), number-average molecular mass (*M*_n_), weight-average molecular mass (*M*_w_), PDI, and the temperature of maximum weight loss (*T*_max_). The model checked the curvature and used a 3-way factor and pure error. For *T*_g_, *M*_n_, *M*_w_, PDI, and *T*_max_, the coefficient of variation was lower than 10.0%, calculated from the replicates at a central point (R17–R19). Table 5 shows the analysis of variance (ANOVA) performed for all of the response variables. For all analyses, the factors were considered statistically significant (SS) for *p* ≤ 0.05, marginally significant (MS) for 0.05 < *p* ≤ 0.10, and nonsignificant (NS) for *p* > 0.10. The detailed results of ANOVA for all response variables are shown in the Supporting Information.

**Table 4:** Degree of Lignin Modification, *T*_g_, and Molar Mass Results^a^

experiment	N_SA_ (mmol g^–1^)^b^	*T*_g_ (°C)^c^	*M*_n_ (g mol^–1^)^d^	*M*_w_ (g mol^–1^)^e^	PDI^f^	*T*_max_ (°C)^g^
kraft lignin		183.3	588	6647	11.31	400.7
R1	1.44	182.3	604	4953	8.20	507.0
R2	1.70	150.8	539	4687	8.69	539.0
R3	0.73	118.5	598	5359	8.96	536.3
R4	0.75	169.9	646	5150	7.97	501.8
R5	0.34	186.3	734	9138	12.45	498.3
R6	0.38	186.1	1248	7916	6.34	510.2
R7	0.94	188.6	699	7249	10.37	513.1
R8	0.48	197.6	1224	8747	7.14	512.2
R9	0.94	206.1	1201	6901	5.74	494.3
R10	0.72	179.4	1376	9697	7.05	520.9
R11	0.76	188.8	1283	8474	6.60	504.2
R12	0.81	193.3	740	7950	10.74	506.1
R13	0.33	181.5	732	7990	10.91	501.1
R14	0.99	164.9	1156	6379	5.52	531.3
R15	0.98	160.3	1115	5896	5.29	523.6
R16	1.48	157.6	1084	6114	5.64	525.3
R17	0.80	177.6	1281	7690	6.00	500.0
R18	0.45	186.6	1288	7761	6.02	492.8
R19	0.69	188.8	1302	8111	6.23	508.6

a Notes: Standard deviation (SD) related to experiments R17, R18, and R19.

b 0.18 mmol g^–1^.

c 5.9 °C.

d 10.7 g·mol^–1^.

e 225.4 g·mol^–1^.

f 0.13 °C.

g 7.91 °C.

**Table 5:** ANOVA Results for All Response Variables

factor	N_SA_^a^	*T*_g_^b^	*M*_n_^c^	*M*_w_^d^
Curvature	NS	NS	SS	SS
temperature (X_1_)	SS	SS	SS	SS
imidazole (X_2_)	NS	MS	SS	MS
time (X_3_)	MS	MS	SS	NS
succinic acid (X_4_)	NS	SS	SS	SS
X_1_·X_2_	NS	NS	SS	NS
X_1_·X_3_	MS	NS	SS	MS
X_1_·X_4_	NS	MS	SS	NS
X_2_·X_3_	NS	NS	SS	NS
X_2_·X_4_	NS	NS	MS	SS
X_3_·X_4_	NS	MS	SS	NS
X_1_·X_2_·X_3_	NS	NS	SS	SS
X_1_·X_2_·X_4_	NS	NS	NS	MS
X_2_·X_3_·X_4_	NS	NS	NS	NS
X_1_·X_3_·X_4_	NS	NS	SS	MS

a R^2^: 0.9580.

b 0.6979.

c 0.9994.

d 0.9813. NS = nonsignificant; SS = statistically significant; MS = marginally significant.

The results presented in Table 4 are particularly intriguing. The highest incorporation of SA groups into the lignin structure in this study was achieved in R17, reaching 1.80 mmol·g^–1^. Lignin’s molecular structure can explain that outcome: the presence of aromatic rings and cross-links creates steric hindrance, making it challenging to introduce new groups.^4,32^ Additionally, esterification reactions naturally reach equilibrium, necessitating the removal of byproducts (water, in this case) or other strategies to shift the reaction toward the esterified product. High N_SA_ values were also observed in R1 and R2 (1.44 and 1.70 mmol·g^–1^, respectively), which were conducted at 160 °C for 15 h. The effects of the temperature (X_1_) and time (X_3_) were deemed significant and MS, respectively, for N_SA_. These parameters play crucial roles in reaction conversion.^33,34^ Interestingly, the amounts of imidazole (X_2_) and succinic acid (X_4_) were insignificant. However, the interaction between temperature and time (X_1_·X_3_) was MS for N_SA_, indicating that an appropriate combination of SA amount, catalyst, temperature, and time is critical for achieving desirable N_SA_ values in shorter reaction times, as seen in R3 (0.73 mmol·g^–1^, 5 h).

**Figure 5:**
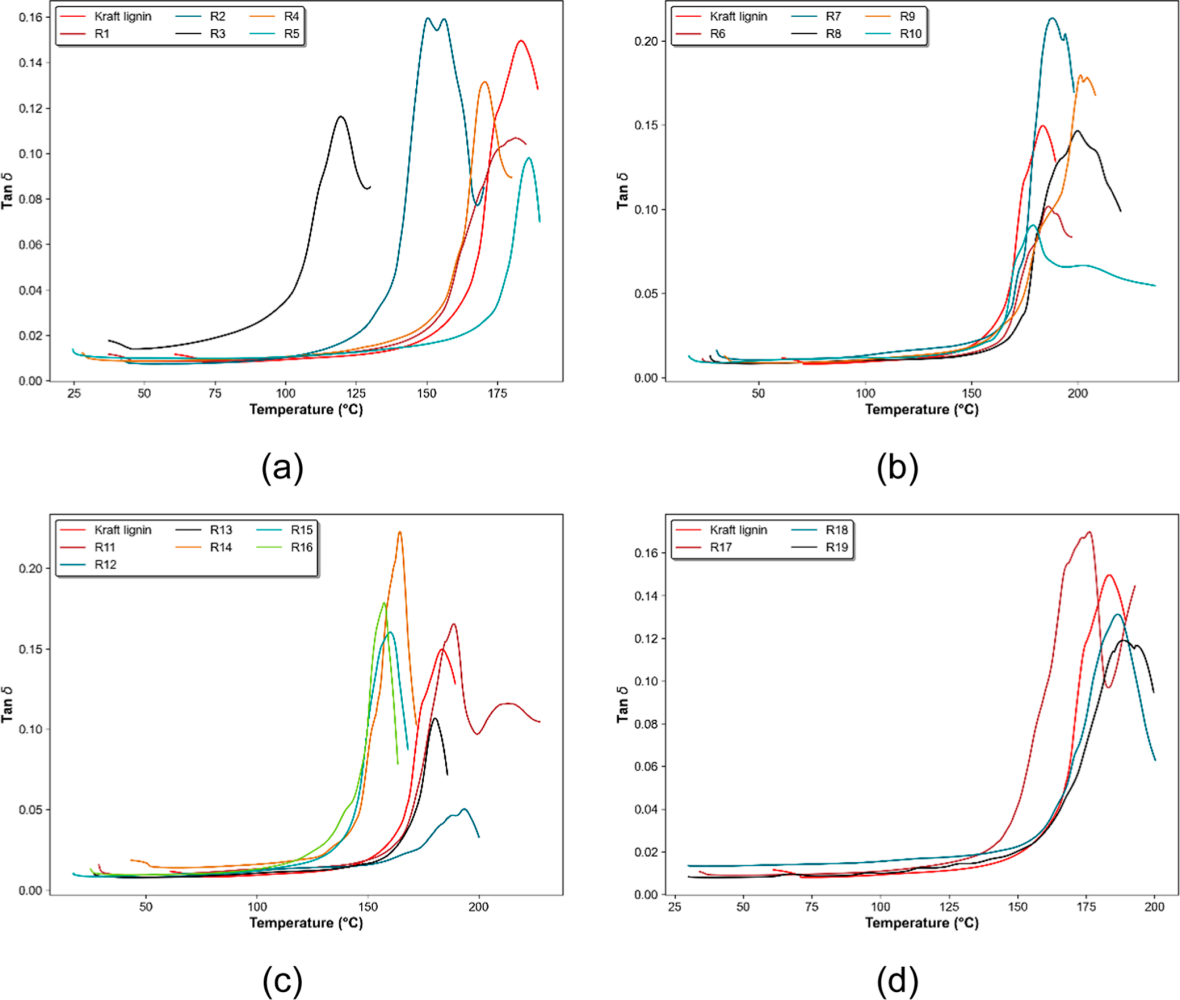
Thermograms showing tan δ of modified lignins. (a) R1–R5; (b) R6–R10; (c) R11–R16; (d) R17–R19.

Tan δ thermograms obtained from DMA analyses are shown in Figure 5.

Multiple factors can influence lignin’s glass transition temperature. Cleavage or condensation side reactions may occur during the reaction step, which leads to a change in conformation of the lignin structure, increasing (or decreasing) the *T*_g_.^35^ In addition, the separation step of the final product can also result in lignin fractionating, which changes the material’s *T*_g_.^36^ In this context, the results presented in Table 4 cannot be exclusively assigned to the chemical modification of lignin.

Some modified lignins showed two peaks (or “shoulders”) in the tan δ thermograms (Figure 5). This may be attributed to differences in segmental mobility of hard and soft segments separately, indicating the formation of a heterogeneous material.^37^ However, as the tan δ thermogram of kraft lignin shows only one peak, this is further evidence of chemical modification of the lignin structure.

The *T*_g_ of modified lignin in all experiments was different from the *T*_g_ of kraft lignin, which can be an indication that the chemical modification reaction has occurred (Table 4). The reaction performed at 160 °C, with 0.13 mol of imidazole and 0.35 mol of SA for 5 h (R3), showed the sharpest reduction in *T*_g_ (118.5 °C), which indicates that it was possible to reduce the *T*_g_ of lignin with short reaction times by adjusting other important factors such as temperature and the amount of SA.

**Figure 6:**
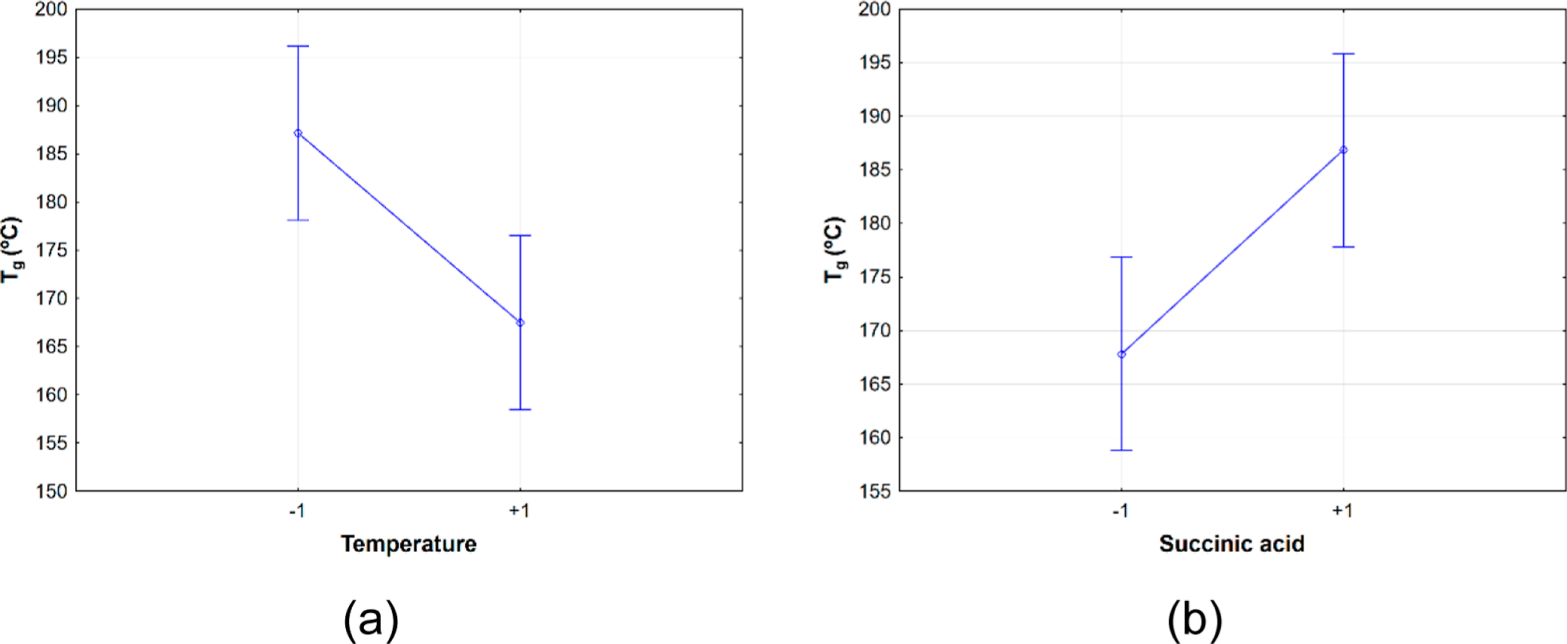
Means plots of *T*_g_ versus reaction temperature (a) and amount of SA (b).

According to Table 5, the temperature (X_1_) and the amount of succinic acid (X_4_) were the only SS variables for *T*_g_. The plot of margninal means shows that *T*_g_ is inversely proportional to the reaction temperature (Figure 6a) and directly proportional to the amount of succinic acid (Figure 6b). In fact, temperature was crucial in chemical reactions as higher temperatures can lead to higher conversions.^38^ In this case, the reaction mechanism includes the consumption of hydroxyl groups and the formation of ester groups, which reduces the hydrogen bonding in the lignin structure and, therefore, the *T*_g_. Also, the amount of SA was directly proportional to the *T*_g_. It can be explained in terms of the interactions between SA molecules and lignin. If the reaction was carried out with an excess of SA, unreacted molecules of SA will be available in the reaction medium. These unreacted molecules can form hydrogen bonds with lignin once there are more hydroxyl groups available from succinic acid molecules, increasing the final product’s *T*_g_. Besides, it is important to highlight that time (X_3_) was MS. An increase in the reaction time can lead to undesired side reactions, such as condensation of aromatic rings. These side reactions can change the conformation of lignin’s structure, leading to higher *T*_g_.

Regarding SEC analyses, it is important to mention that all experiments increased the *M*_n_ of lignin, except R2. Another significant result was that, except for R5, PDI decreased in all experiments, which indicates a narrower PDI for modified lignin. Narrow PDI shows some advantages, including increased mechanical strength, toughness, and stress crack resistance of lignin.^39,40^ Furthermore, as previously discussed, the separation process of the final product may induce lignin fractionation, thereby altering the material’s PDI as the *T*_g_.

As mentioned before, reactions involving lignin often exhibit unpredictable behavior, because of its complex chemical structure. The intramolecular ether linkages in lignin, primarily aril-ether β-O-4 and α-O-4 bonds, are susceptible to cleavage at elevated temperatures because these linkages have the lowest bond dissociation energy.^41^ Once cleaved, lignin undergoes a rearomatization process, leading to the formation of condensed side reactions. As the C–O bonds in the lignin structure are consumed with increasing temperature, less reactive C–C sites are formed.^32^

Moreover, the polarity of the medium affects the molecule’s conformation and, consequently, the availability of hydroxyl groups. Concurrently, changes in lignin’s intermolecular and intramolecular structures impact the reaction process. However, beyond the scope of this study, future investigations may evaluate the thermodynamic and kinetic contexts of these reactions.

These phenomena occur simultaneously, and various reaction conditions—such as temperature, reagent concentration, and time—significantly influence the final product. This interplay explains the apparent lack of reaction control when analyzing molecular weight distribution responses. Thus, this underscores the importance of employing a factorial design of experiments to systematically assess the overall process as a function of these events.

The results shown in Table 5 indicate that all factors and their interactions influence *M*_n_ except for the combination of imidazole (X_2_) and SA (X_4_) (MS) and for the interaction between imidazole, time (X_3_), and SA (X_4_) (nonsignificant). Besides, ANOVA also shows that the curvature was SS for the proposed model. However, as the factorial design of experiments used in this study includes only two levels, it was impossible to obtain a model with quadratic terms. It was possible to see that the main effect on *M*_n_ was the synergetic effect between the temperature (X_1_) and time (X_3_). The literature shows how these two variables strongly affect the polymer properties.^33,42,43^ However, time and *M*_n_ were directly proportional, while temperature and *M*_n_ were inversely proportional. It suggests that higher temperatures favor cleavage reactions in the lignin structure, reducing the *M*_n_. Temperatures exceeding 100 °C can trigger the release of water from lignin’s structure because of the cracking of aliphatic hydroxyl groups in its lateral chains. Furthermore, at temperatures above 150 °C, a rupture of specific C–C linkages in the alkyl chains may be observed, leading to the liberation of CO and CO_2_.^44,45^ Nonetheless, the particular reaction conditions could change the range of cleavage noted in the literature. As expected, the amount of SA also increases *M*_n_ as new chains are formed during the reaction. Nevertheless, results in Table 3 suggest that this effect can be limited because of the consumption of hydroxyl groups from lignin and, therefore, the increase in the unreacted amount of SA.

When *M*_w_ was the dependent variable, the temperature seemed to be the most important factor. Surprisingly, the temperature and *M*_w_ were inversely proportional, which means higher temperatures may favor side reactions or even cleavage in the lignin structure. As discussed for *M*_n_, the curvature was SS according to ANOVA, which means that the influence of the factors on *M*_w_ was better described with quadratic terms. According to Table 4, time was not important for *M*_w_, although the combination of this factor with the temperature (X_1_·X_3_) was MS.

As expected, all factors and their synergetic effects were significant for PDI as this property depends on *M*_n_ and *M*_w_. Temperature, time, and the synergetic effect between these two variables were the most important factors, producing lignin with a broader PDI when these two factors are combined. Once again, the curvature was also important and a central composite design was required to evaluate the best model to describe the observations adequately.

**Figure 7:**
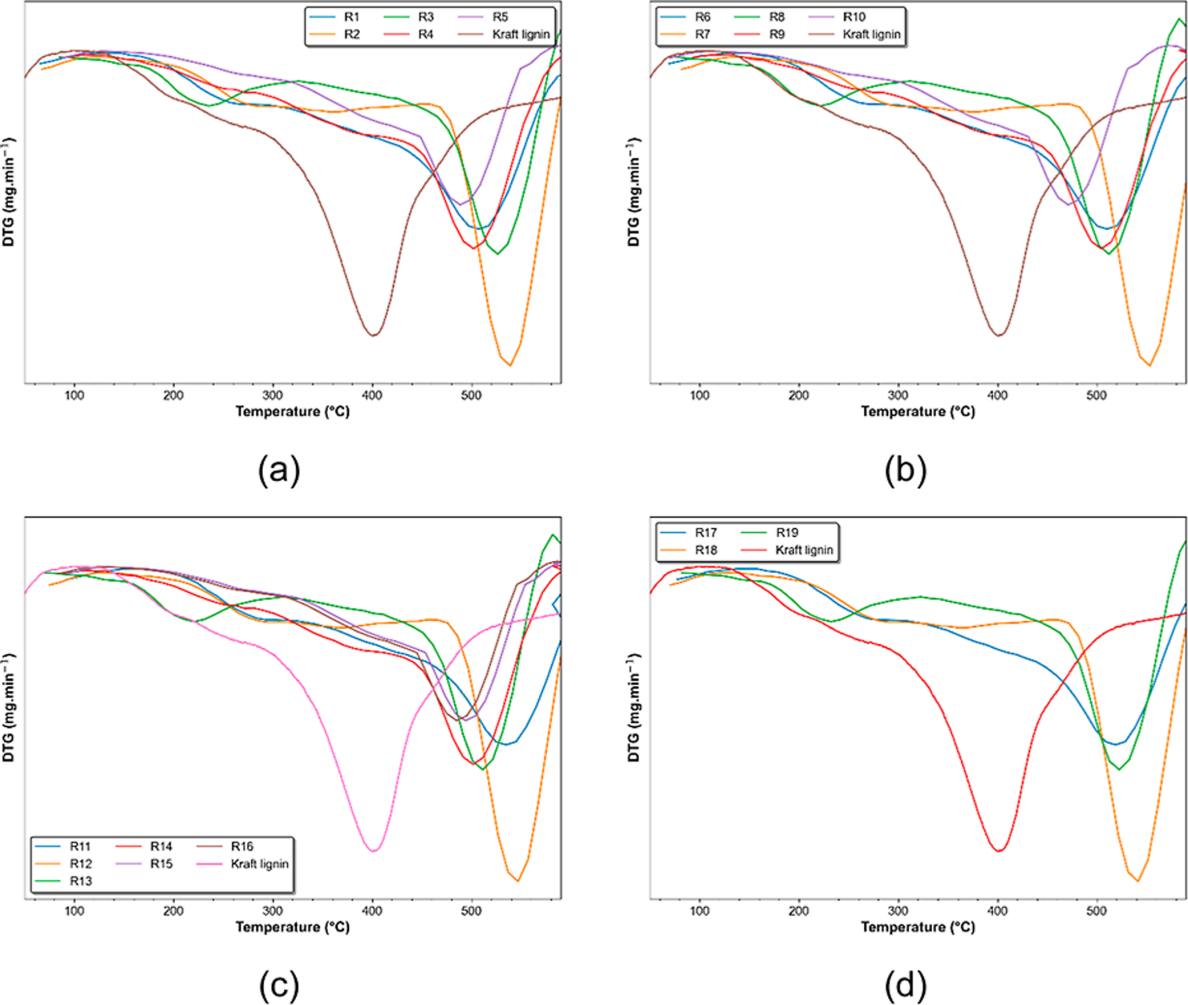
DTG thermograms of modified lignins. (a) R1–R5; (b) R6–R10; (c) R11–R16; (d) R17–R19.

DTG thermograms are shown in Figure 7.

DTG thermograms also indicated the chemical modification of the lignin structure. The peak of maximum weight loss of kraft lignin is at 400 °C, while the peak of maximum weight loss of all modified lignins was near 500 °C in all experiments. It suggests that the chemical modification of the lignin structure was achieved and that modified lignin shows better thermal stability than kraft lignin.

## 4 Conclusions

The chemical modification of kraft lignin with SA was performed under varying temperature conditions, reaction times, and amounts of SA and the catalyst (imidazole). The modification of the lignin structure was confirmed through spectroscopic analyses. FTIR spectra showed an increase in the C=O stretching band of esters (1725 cm^–1^), suggesting that SA may have reacted with the lignin structure or may be present as an unreacted impurity. ^1^H NMR and ^31^P NMR analyses confirmed the proposed mechanism for lignin modification with SA, with the former being used to quantify the impurity content under the different conditions. DTG thermograms demonstrated that the experimental conditions led to the formation of more thermally stable lignin. Statistical ANOVA revealed that temperature was the critical factor influencing all response variables, affecting both extent of the reaction and the properties of the modified lignin. Additionally, an ideal condition (160 °C, 0.13 mol imidazole, 0.35 mol SA, and 5 h reaction time) was identified, combining high temperature with low reaction times and minimal amounts of SA and the catalyst (R3). Under these conditions, the modified lignin exhibited the lowest glass transition temperature (*T*_g_) and a narrower molar mass distribution, suggesting its potential use as a precursor for carbon fiber production and other applications requiring plasticization. Specifically, this modified lignin could serve as a functionalized or esterified intermediate for lignin-based resins. Furthermore, once the reaction is successfully completed, yield investigations may be conducted, emphasizing the process’s sustainability.

## Supporting Information Available

ANOVA results for the degree of modification of lignin, *T*_g_, Mn, Mw, PDI, ^1^H NMR spectra, content of free SA, and SEC chromatograms of kraft lignin and R1–R19 samples (PDF)
