# Supplementary material for: Chemical Modification of Softwood Kraft Lignin with Succinic Acid
Source: ACS Omega. 2024 Dec 19;9(52):50945–56. doi: 10.1021/acsomega.4c03127 (PMC11696390; doi:10.1021/acsomega.4c03127)

Chemical Modification of Softwood Kraft Lignin with Succinic Acid

Gabriel Resende†*, Gustavo D. Azevedo†, Felipe Souto† and Veronica Calado†

†Programa de Engenharia de Processos Químicos e Bioquímicos, Escola de Química, Centro de Tecnologia, Universidade Federal do Rio de Janeiro, Av. Athos da Silveira Ramos, 149, Bloco E, Ilha do Fundão, Rio de Janeiro, 21941-909, Brasil

Table S1. ANOVA results for the degree of modification of lignin.

Factor	Sum of squares	Degrees of freedom	Mean sum of squares	F-value	p-value
Curvature	0,111308	1	0,111308	3,39297	0,206812
Temperature (X_1)	1,141984	1	1,141984	34,81086	0,027545
Imidazole (X_2)	0,024503	1	0,024503	0,74692	0,478549
Time (X_3)	0,284454	1	0,284454	8,67095	0,098570
Succinic acid (X_4)	0,002034	1	0,002034	0,06202	0,826576
X_1•X_3	0,334616	1	0,334616	10,20003	0,085634
Lack of fit	0,045714	1	0,045714	1,39349	0,359191

R^2: 0.9580 Adjusted-R^2: 0.7484 Pure error: 0.0328

Table S2. ANOVA results for T_g.

**Factor**	**Sum of squares**	**Degrees of freedom**	**Mean sum of squares**	**F-value**	**p-value**
Temperature (X_1)	1552.557	1	1552.557	44.20318	0.021883
Imidazole (X_2)	314.264	1	314.264	8.94748	0.095948
Time (X_3)	618.144	1	618.144	17.59930	0.052395
Succinic acid (X_4)	1452.563	1	1452.563	41.35622	0.023337
X_1•X_4	369.312	1	369.312	10.51477	0.083382
X_3•X_4	486.975	1	486.975	13.86475	0.065155
Lack of fit	2005.356	10	200.536	5.70949	0.158146

R^2: 0.6979. Adjusted-R^2: 0.5468. Pure error: 35.1232

Table S3. ANOVA for M_n.

Factor	Sum of squares	Degrees of freedom	Mean sum of squares	F-value	p-value
Curvature	316849	1	316848.7	2771.271	0.000361
Temperature (X_1)	74666	1	74665.6	653.052	0.001528
Imidazole (X_2)	49395	1	49395.1	432.027	0.002307
Time (X_3)	262400	1	262400.1	2295.044	0.000435
Succinic acid (X_4)	128702	1	128701.6	1125.670	0.000887
X_1•X_2	67211	1	67210.6	587.847	0.001697
X_1•X_3	360901	1	360900.6	3156.565	0.000317
X_1•X_4	60393	1	60393.1	528.219	0.001888
X_2•X_3	71690	1	71690.1	627.027	0.001591
X_2•X_4	1008	1	1008.1	8.817	0.097169
X_3•X_4	38711	1	38710.6	338.576	0.002941
X_1•X_2•X_3	120930	1	120930.1	1057.697	0.000944
X_1•X_3•X_4	73306	1	73305.6	641.157	0.001556
Lack of fit	738	3	245.9	2.151	0.333033

R^2: 0.9994. Adjusted-R^2: 0.9979. Pure error: 114.33

Table S4. ANOVA for M_w

**Factor**	**Sum of squares**	**Degrees of freedom**	**Mean sum of squares**	**F-value**	**p-value**
Curvature	1684225	1	1684225	33.1560	0.028861
Temperature (X_1)	29490330	1	29490330	580.5526	0.001718
Imidazole (X_2)	771762	1	771762	15.1931	0.059961
Succinic acid (X_4)	940900	1	940900	18.5227	0.049975
X_1•X_3	846400	1	846400	16.6624	0.055102
X_2•X_4	1688700	1	1688700	33.2441	0.028788
X_1•X_2•X_3	2168256	1	2168256	42.6847	0.022635
X_1•X_2•X_4	478864	1	478864	9.4270	0.091718
X_1•X_3•X_4	861184	1	861184	16.9534	0.054231
Lack of fit	639111	7	91302	1.7974	0.403297

R^2: 0.9813. Adjusted-R^2: 0.9623. Pure error: 50797.

Table S5. ANOVA for PDI

Factor	Sum of squares	Degrees of freedom	Mean sum of squares	F-value	p-value
Curvature	9.03853	1	9.03853	580.349	0.001719
Temperature (X_1)	15.20583	1	15.20583	976.341	0.001023
Imidazole (X_2)	0.53969	1	0.53969	34.652	0.027666
Time (X_3)	18.59887	1	18.59887	1194.203	0.000836
Succinic acid (X_4)	3.53054	1	3.53054	226.690	0.004382
$X_1 \cdot X_2$	3.49159	1	3.49159	224.189	0.004431
$X_1 \cdot X_3$	18.97526	1	18.97526	1218.370	0.000820
$X_1 \cdot X_4$	1.90336	1	1.90336	122.212	0.008083
$X_2 \cdot X_3$	3.81718	1	3.81718	245.095	0.004055
$X_2 \cdot X_4$	1.30589	1	1.30589	83.849	0.011717
$X_3 \cdot X_4$	2.72806	1	2.72806	175.164	0.005660
$X_1 \bullet X_2 \bullet X_3$	1.39406	1	1.39406	89.510	0.010988
$X_1 \bullet X_2 \bullet X_4$	0.36681	1	0.36681	23.553	0.039932
$X_1 \bullet X_3 \bullet X_4$	0.97902	1	0.97902	62.861	0.015538
$X_2 \bullet X_3 \bullet X_4$	0.49926	1	0.49926	32.056	0.029807
Lack of fit	0.04156	1	0.04156	2.669	0.243948

R^2: 0.9991. Adjusted-R^2: 0.9947. Pure error: 0.0156

Table S6. Content of free succinic acid

Sample	Free succinic acid ($mmol.g^{-1}$)
R1	0,16
R2	0,24
R3	0,29
R4	0,14
R5	0,07
R6	0,11
R7	0,25
R8	0,07
R9	0,19
R10	0,32
R11	0,21
R12	0,18
R13	0,12
R14	0,21
R15	0,16
R16	0,18
R17	0,23
R18	0,15
R19	0,19

Figure S1. ^{1}H-NMR spectra of kraft lignin, R1, R2, and R3

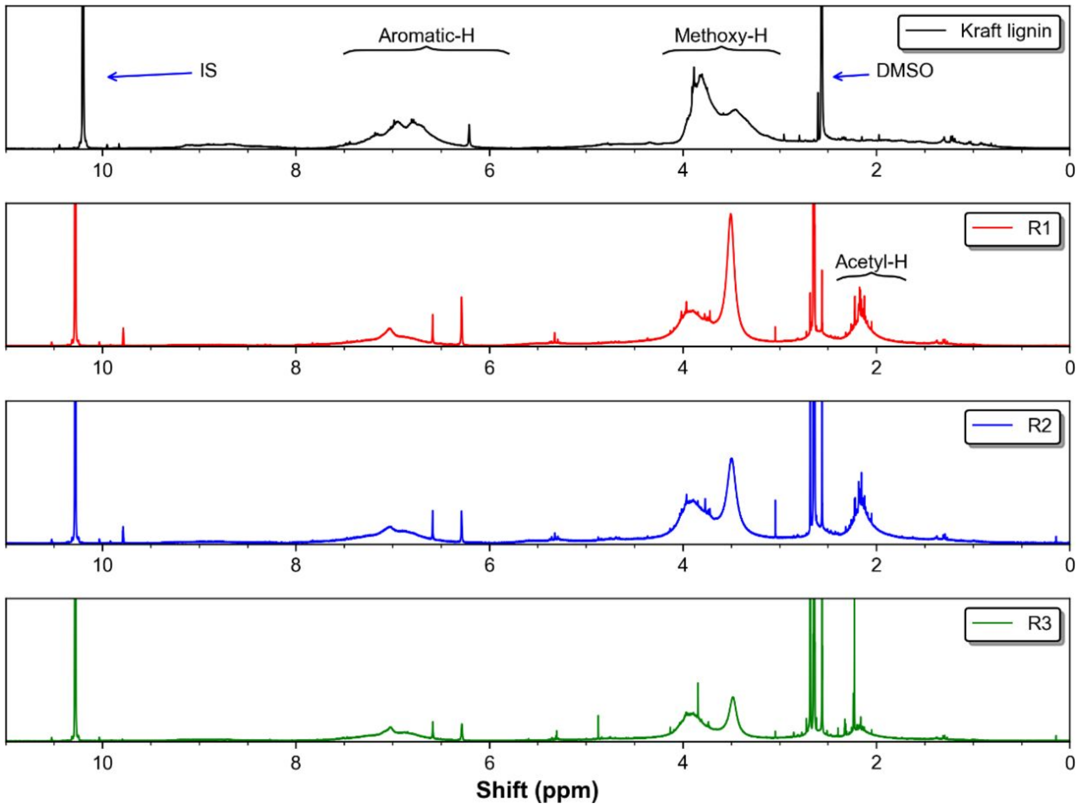

Figure S2. ^{1}H-NMR spectra of R4, R5, R6 and R7

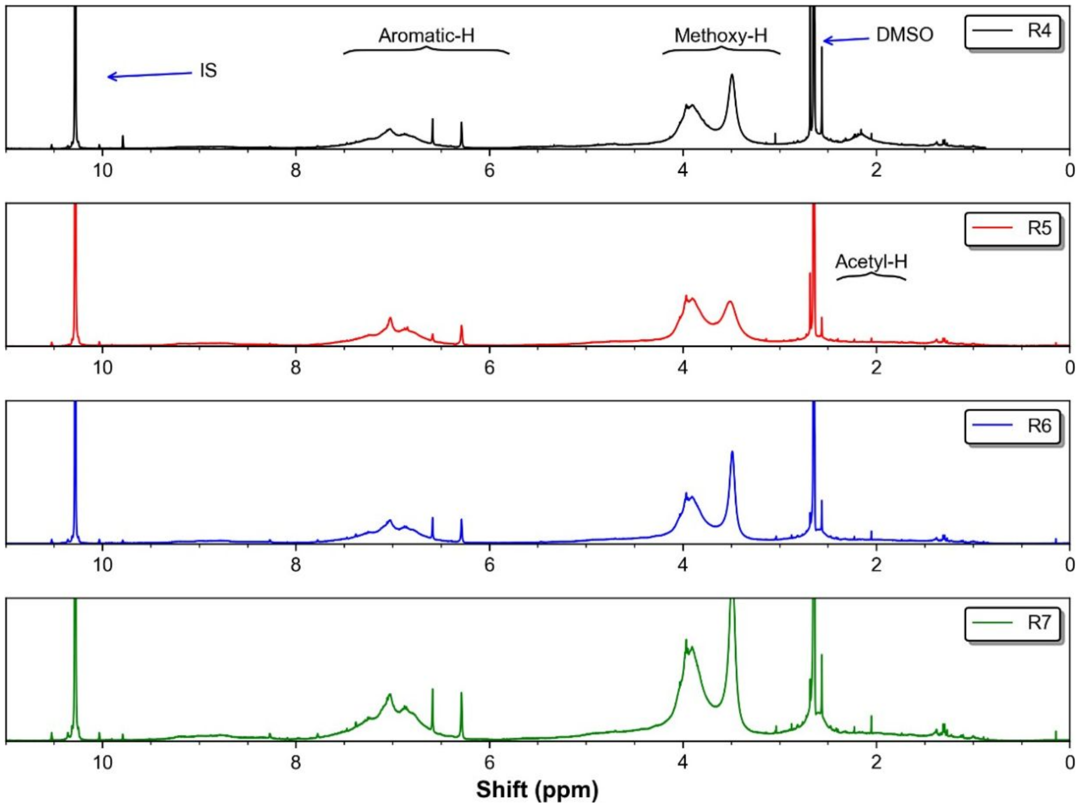

Figure S3. ^{1}H-NMR spectra of R8, R9, R10 and R11

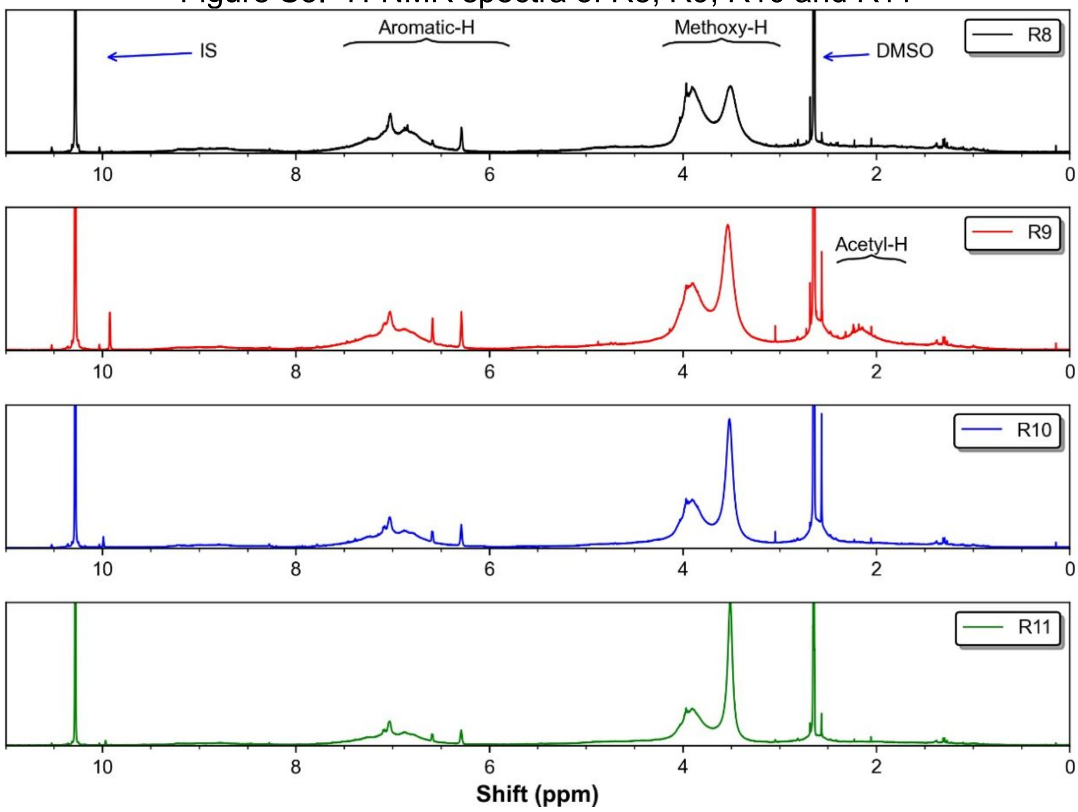

Figure S4. ^{1}H-NMR spectra of R12, R13, R14 and R15

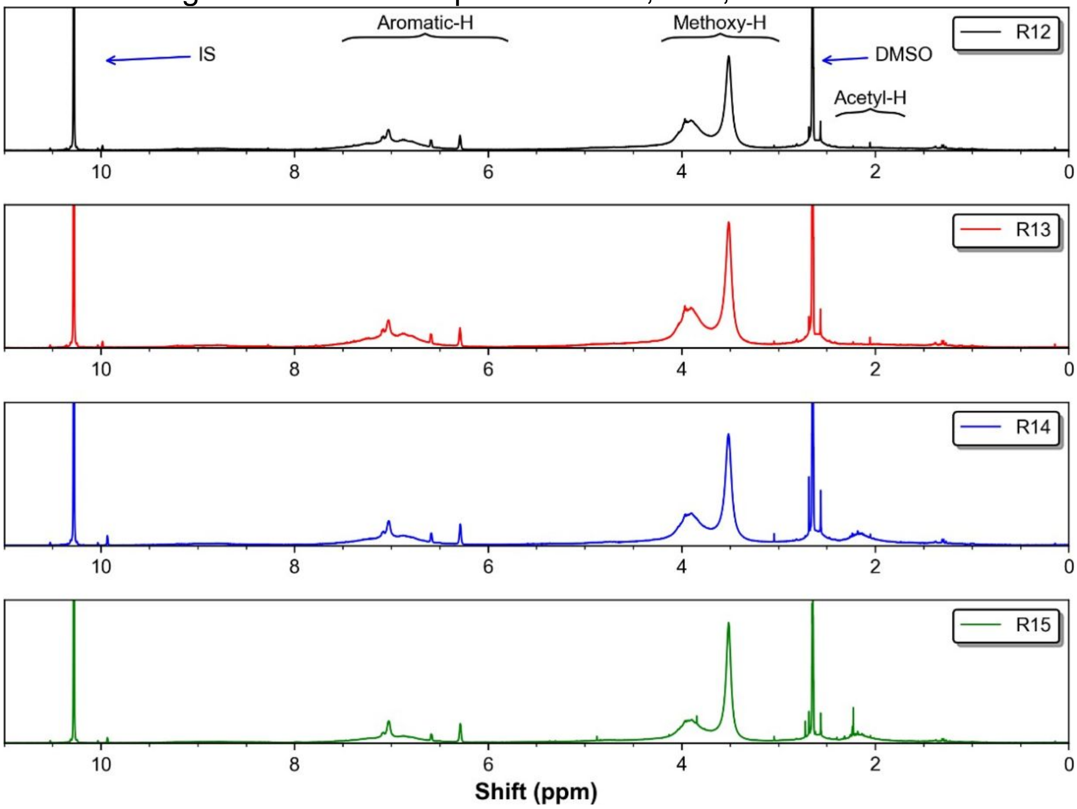

Figure S5. ^{1}H-NMR spectra of R16, R17, R18 and R19

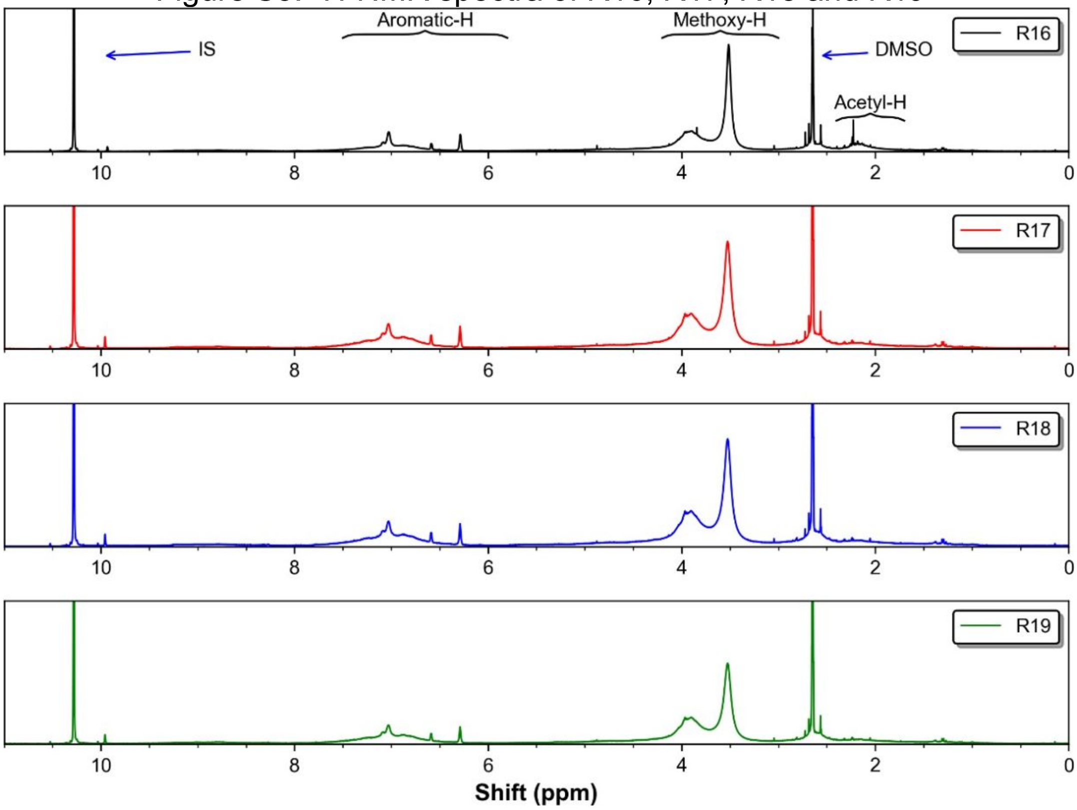

Figure S6. SEC chromatograms of kraft lignin, R1, R2 and R3

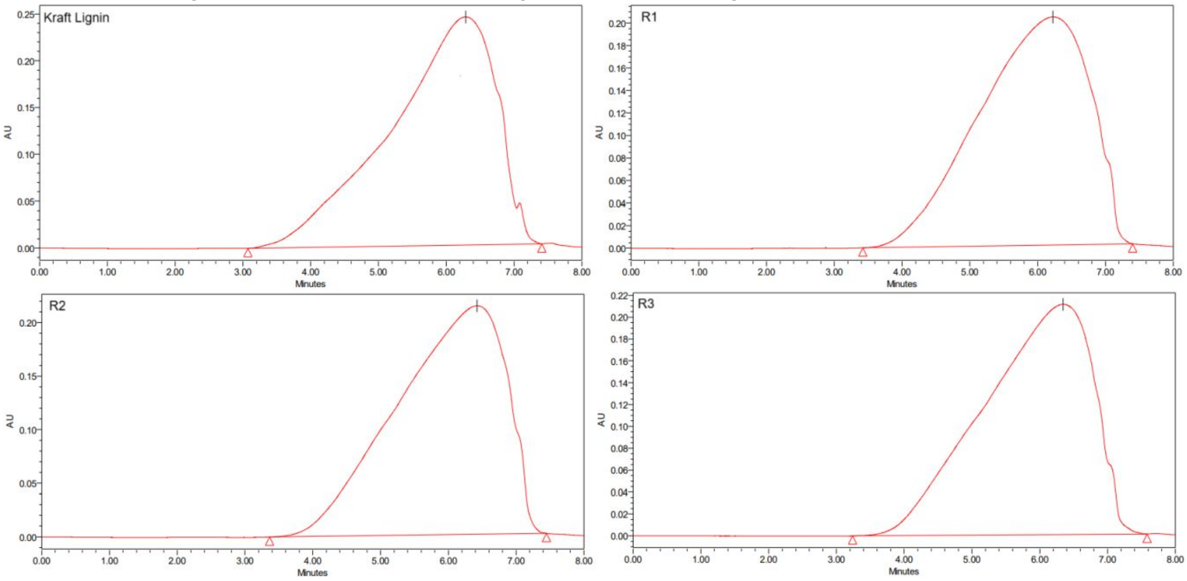

Figure S7. SEC chromatograms of R4, R5, R6 and R7

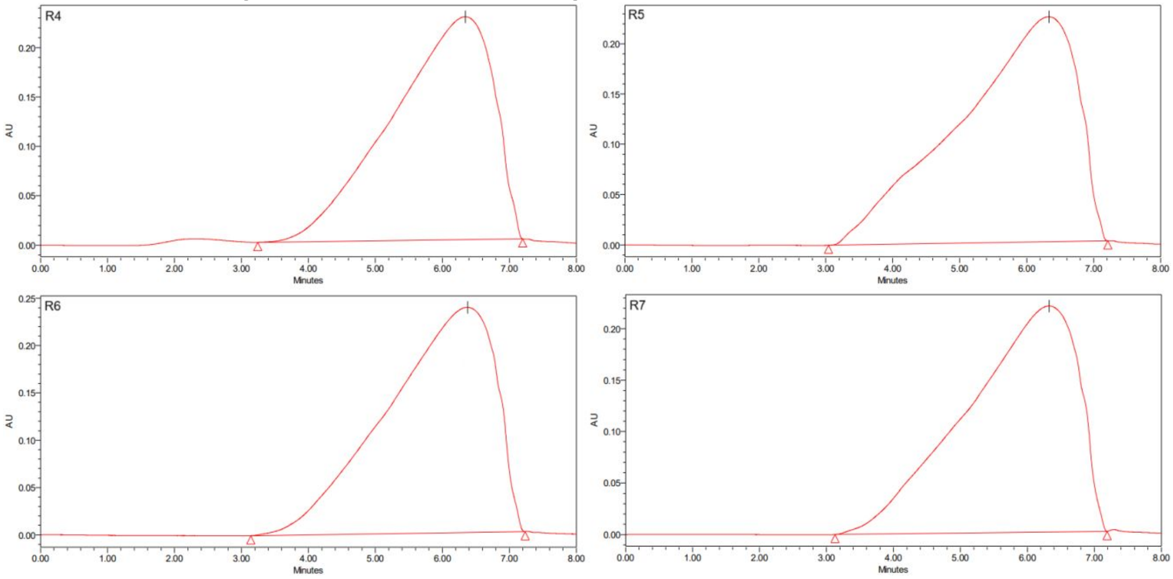

Figure S8. SEC chromatograms of R8, R9, R10 and R11

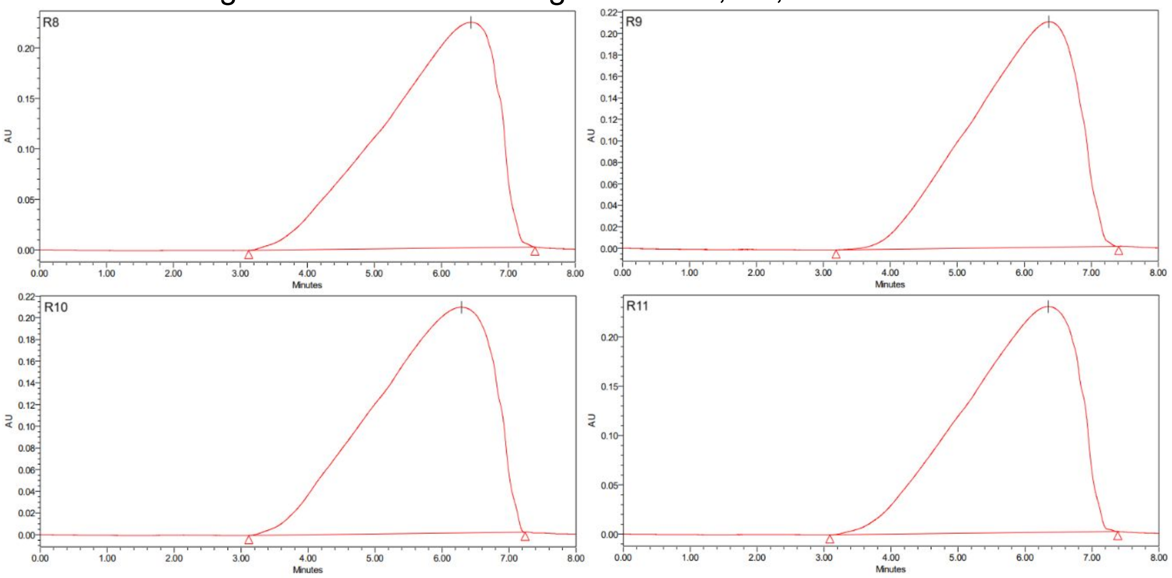

Figure S9. SEC chromatograms of R12, R13, R14 and R15

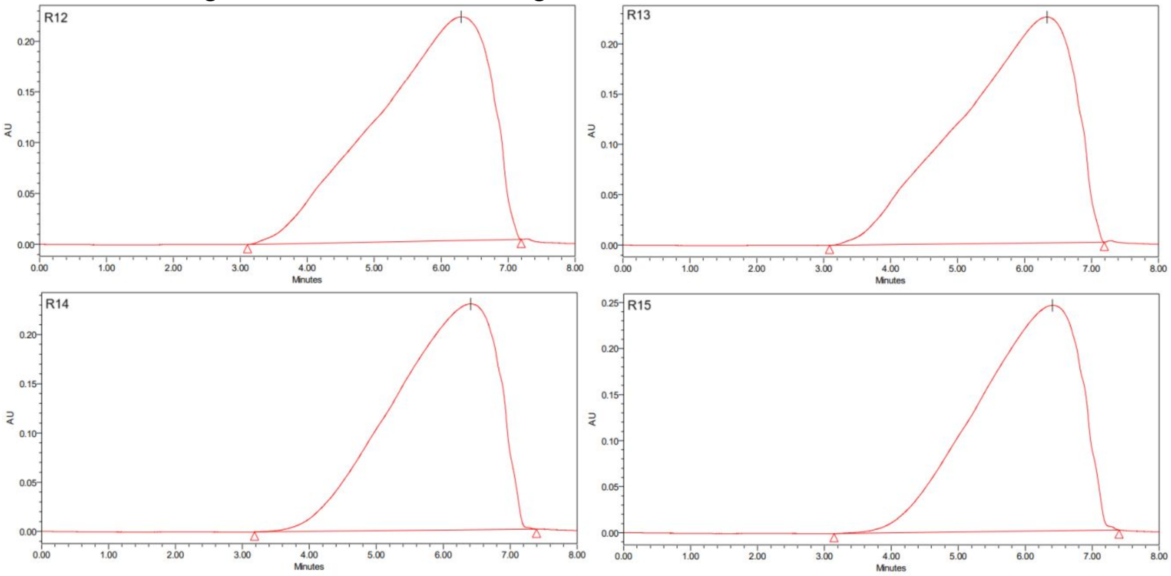

Figure S10. SEC chromatograms of R16, R17, R18 and R19

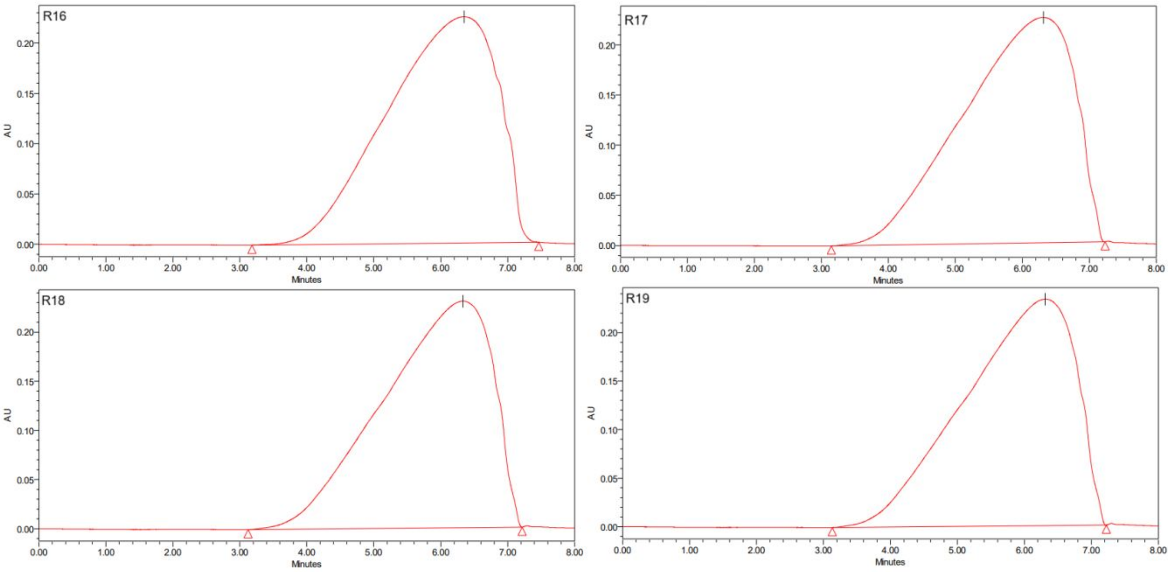

Supplement: Supplementary file 1 — ao4c03127_si_001.pdf [file ao4c03127_si_001.pdf]
